# Integrative molecular profiling identifies two molecularly and clinically distinct subtypes of blastic plasmacytoid dendritic cell neoplasm

**DOI:** 10.1038/s41408-022-00699-1

**Published:** 2022-07-04

**Authors:** Axel Künstner, Julian Schwarting, Hanno M. Witte, Veronica Bernard, Stephanie Stölting, Kathrin Kusch, Kumar Nagarathinam, Nikolas von Bubnoff, Eva Maria Murga Penas, Hartmut Merz, Hauke Busch, Alfred C. Feller, Niklas Gebauer

**Affiliations:** 1 University Cancer Center Schleswig-Holstein, University Hospital of Schleswig-Holstein, Campus Lübeck, 23538 Lübeck, Germany; 2 Medical Systems Biology Group, University of Lübeck, Ratzeburger Allee 160, 23538 Lübeck, Germany; 3 Institute for Cardiogenetics, University of Lübeck, Ratzeburger Allee 160, 23538 Lübeck, Germany; 4 Department of Hematology and Oncology, University Hospital of Schleswig-Holstein, Campus Lübeck, Ratzeburger Allee 160, 23538 Lübeck, Germany; 5 Hämatopathologie Lübeck, Reference Centre for Lymph Node Pathology and Hematopathology, Maria-Goeppert-Straße 9a, 23562 Lübeck, Germany; 6 Department of Hematology and Oncology, Federal Armed Forces Hospital Ulm, Oberer Eselsberg 40, 89081 Ulm, Germany; 7 Institute of Biochemistry, University of Lübeck, Ratzeburger Allee 160, 23538 Lübeck, Germany; 8 Institute of Human Genetics, University Hospital of Schleswig-Holstein, Campus Kiel, Schwanenweg 24, 24105 Kiel, Germany

**Keywords:** Cancer genetics, Cancer genomics, Leukaemia

Dear Editor,

Blastic plasmacytoid dendritic cell neoplasm (BPDCN) is a rare and aggressive blood cancer. In the era of conventional chemotherapy prognosis was poor with high rates of relapse and refractory disease despite consolidating allogenic or autologous stem cell transplantation [1]. Therapy with curative intent was therefore reserved for young and otherwise healthy patients, until the introduction of tagraxofusp, a CD123-directed cytotoxin conjugate, which recently demonstrated high clinical efficacy across all age groups [2]. Median age at diagnosis lies within the seventh decennium and a male predominance is observed [3]. Cutaneous involvement often precedes bone marrow infiltration and dissemination into lymph nodes or other organs while primary leukemic disease is rare. The discovery of its predominant cellular descent from CD56^+^ plasmacytoid dendritic cells (pDC) led to the recognition of BPDCN as an independent entity within the WHO classification of myeloid neoplasms [4]. Although the characteristic immunophenotype facilitates specific diagnosis, differentiation from acute myeloid leukemia (AML) can be challenging. Recent observations proposed a subset of BPDCN to originate from AXL1^+^ SIGLEC6^+^ DCs (AS-DCs), suggesting a heterogeneous cellular ontogeny [5]. Panel and whole-exome sequencing (WES) on small cohorts and transcriptome sequencing (RNA-seq) of selected patients have reported a limited number of potential genetic drivers and transcriptional mechanisms underlying BPDCN [6, 7]. Syn- and metachronous myeloid neoplasms (CMML, AML and MDS) have been reported in up to 20% of cases [8]. This is reflected in myeloid mutational features of BPDCN, comprising mutations in epigenetic regulation (*TET2*, *ASXL1*, *EZH2*), RAS signaling (*NRAS*, *KRAS*), splicing (*ZRSR2*, *SF3B1*) and tumor suppressors (TSGs; *TP53*, *ATM*). Recently, mutations in epigenetic regulators were shown to be a recurrent feature of clonal hematopoiesis, underlying BPDCN [9].

We collected 47 diagnostic cases of BPDCN with sufficient FFPE tissue samples for molecular studies (mean/median age 69.0/74.0 years; range 15–91 years; located in skin (*n* = 25), lymph node (*n* = 11), bone marrow (*n* = 9) and others (*n* = 2)). For details on case selection, extraction of nucleic acids, WES, RNA-seq, somatic copy number aberration (SCNA) analysis and data processing please see supplementary materials and methods. Baseline clinicopathological characteristics are summarized in Supplementary Tables 1 and 2. In this hitherto most comprehensive, paired genomic and transcriptomic study of BPDCN, supplemented by SCNA analysis, we made three essential observations. First, employing MutSigCV we identified 41 significant candidate driver genes (*p* < 0.001; 20 genes with *q* < 0.1; Supplementary Table 5) and thereby established a precision oncology roadmap of targetable vulnerabilities. Across the cohort, *TET2* was the gene most frequently mutated, as expected, followed by *KMT2D* and *EP300* (Fig. 1A). The list of significant candidate driver genes included several genes previously implicated in BPDCN and further expanded on these [7, 10]. Subsequent gene set enrichment analysis delineated a significant impact of oncogenic mutations on the epigenetic regulation of gene expression (95.7% of cases; *TET2*, *DNMT3A*, *KMT2D*, *SETD2*, *IDH2*), RTK-RAS (93.6%; *NRAS*, *MET*, *EGFR*), NOTCH (76.6%; *NOTCH2*, *CREBBP*, *EP300*) and WNT signaling pathways (59.6%; *CTNNB1*, *MED12*) (Supplementary Fig. 1). Several therapeutically actionable vulnerabilities were observed, including activating receptors (e.g., *EGFR*) and activating GTPases (e.g., *NRAS*) (Supplementary Fig. 2). Tyrosine kinases such as *MET*, *PDGFRA, ALK* and methyltransferase enzymes, including *EZH2* or TSGs like *CDKN2A*, pose viable targets for molecularly tailored therapy approaches. We further describe a subgroup of five patients with MSI^high^, suggesting an immune checkpoint inhibitor treatment [11]. This is emphasized by recurrent deletions and reduced expression of *MLH1* and deleterious mutations of *MSH6* [12, 13]. Annotations and functional implications of all reported mutations are summarized in Supplementary Table 6. A pair-wise Fisher’s exact test for mutual exclusivity or co-occurrence of mutations, found evidence for mutual exclusivity in *CIC* and *MET* correlated with *NRAS* mutations (Supplementary Fig. 3A). Moreover, several, significant combinations of co-occurrences were observed, including *MPL* and *NOTCH2*, *SETD2*, and *TSC1* as well as *EGFR* and *EP300* alongside *SETD2* (Supplementary Fig. 3B).

**Fig. 1:**
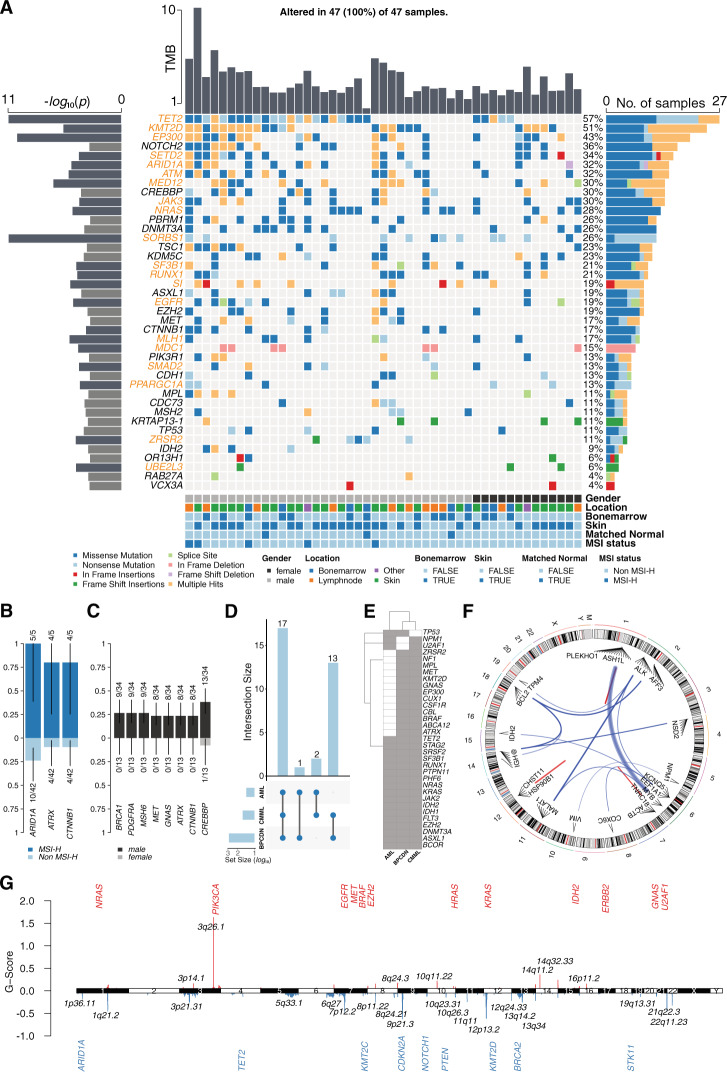
Molecular landscape in BPDCN. **A** Oncoplot showing potential driver genes inferred by MutSigCV with tumor mutational burden (TMB; upper bar plot), −log_10_-transformed *p* values (left bar; orange gene names *q* < 0.1, black gene names *p* < 0.001) and number of affected samples (right bar). In total 13,908 presumably deleterious mutations, affecting 4507 genes were observed. SNVs and indels comprised 39.8% of these mutations (5532), of which 4524 were missense (81.8%), 289 nonsense (5.2%) and 468 indel mutations (8.5%). Mutation types are color-coded, and covariates are shown below for each sample (covariate “Other” refers to samples with tissue affected other than skin or bone marrow). Genes significantly enriched in MSI-H samples (**B**) and with a male mutation bias (**C**); the number of affected samples and the total number of samples are given and the scale on the *y*-axis denotes the proportion of mutated samples. **D** UpSet plot showing the overlap between BPDCN samples (*n* = 47, this study, MutSigCV genes selected), CMML (*n* = 76, Tyner et al.) and AML (*n* = 672, Tyner et al.) for genes mutated in at least two samples per data set (only overlapping genes are shown); **E** overlapping genes between the three data sets for genes mutated in at least two entities; **F** known cancer and *MYB* fusion identified in BPDCN samples with respect to their genomic location; red links indicate intra-chromosomal fusions, blue links indicate inter-chromosomal fusions, respectively. Link width correlates with the number of reads supporting the fusion event; **G** location of SCNAs along the genome and gistic G-scores (G = Frequency × Amplitude; red bars denote gains and blue bars losses; gene names refer to affected oncogenes and tumor-suppressor genes within identified regions).

Secondly, we extend the understanding of the BPDCN molecular landscape in relation to its neighboring entities and expose it to be significantly shaped by micro-satellite-instability (MSI) status, gender and age. A significant enrichment of mutations affecting *ARID1A*, *ATRX*, and *CTNNB1* in MSI^high^ cases was observed while elderly patients were enriched for *DNMT3A* and *TET2* mutations. (Fig. 1B and Supplementary Fig. 4). Beyond the recently described sex-biased implications of *ZRSR2* mutations in male BPDCN patients, we detected an additional, enrichment of mutations affecting *ATRX* and *CTNNB1* alongside other oncogenic drivers of BPDCN pathogenesis including *BRCA1*, *PDGFRA*, *MSH6*, *MET*, *GNAS*, and *CREBBP* in male patients (Fig. 1C). We further compared our results with TCGA AML (*n* = 672) and CMML (*n* = 76) samples. Apparently, CMML is most closely related to BPDCN, with shared mutational drivers including *BRAF*, *CSFR1*, *EP300*, *MET* and *ZRSR2*. In addition, several mutations co-occur between all three entities including *TET2*, *SRSF2*, *SF3B1*, *NRAS*, *KRAS*, and *IDH1/2*. Only *TP53* mutations were found to be an exclusive commonality between BPDCN and AML but not CMML, potentially reflecting the aggressive nature of the former two (Fig. 1D, E). Further, we detected five recurrent SCNAs classified as pathogenic (e.g., del3p21.31 resulting in a loss of *SETD2* in 11 patients and del10q23.2 resulting in a loss of *PTEN* in five patients) and 12 SCNAs as likely pathogenic according to X-CNV. The genes within these SCNAs recurrently led to the loss of tumor-suppressor genes (TSGs; *CDKN2A*, *NOTCH1*, *RB1*, and *BRCA2*) and copy number gains in oncogenes (*IDH2*, *U2AF1*, *MET*, and *EZH2*) (Fig. 1G and Supplementary Fig. 5 and Supplementary Tables 7 and 8). From RNA-seq we extracted gene expression data and compared these with peripheral blood pDCs (CD45^+^ CD123^+^ BDCA2^+^ CD3^−^) from healthy donors [14]. Differential gene expression analysis unveiled results similar to those obtained by Togami et al., including upregulation of *BCL2*, *MYB*, and others [7]. Given the more comprehensive cohort analyzed in the current project, we additionally observed an upregulation of oncogenes such as *PDGFRA/B*, *EGFR*, *FGFR1*, and others as well as a down-regulation of inflammatory mediators such as *IL2*, *IL22*, and *CXCL8*. Despite the results from our MutSigCV analysis in which *PDGFRA* mutations were shown to be events of only borderline significance (*p* = 0.05), we found a pathobiological relevance of these mutations reflected in its simultaneous significant upregulation, contributing to the phenotype of BPDCN. PDGF signaling, NCAM1 interactions and cell cycle accelerators were over-expressed. In addition, several processes associated with interactions with the extracellular matrix as a recurrent feature in BPDCN were significantly upregulated (Supplementary Fig. 6). Combining the SCNA and RNA-seq data validated the copy number loss of several TSGs including *CDKN2A*, *KMT2D*, and *TP53* on the level of gene expression (Supplementary Fig. 7). A subsequent assessment regarding the impact of significant gene mutations on RNA-seq derived profiles identified high-confidence “*trans*-effects” for *TP53*, *RUNX1* and *CBL* which seem to shape the malignant phenotype in BPDCN. (Supplementary Fig. 8). We identified expected fusion events in BPDCN and successfully validated all detected *MYB* fusions via FISH (Fig. 1F and Supplementary Fig. 9 and Supplementary Table 9).

Thirdly, deconvolution of our bulk gene expression profiles using single-cell transcriptome data predicted abundances of cell types within the mixed cell population of our BPDCN biopsies, independent of localization or tumor cell content/purity. We focused on the distribution of dendritic cell and monocyte subtypes for each case using signatures from scRNA-seq data [15]. Subsequently, hierarchical clustering identified two distinct subpopulations within our cohort, in which pDCs, common DCs (DC1 and DC2) and different monocyte subtypes were prevalent at variable frequencies. A typical pDC-derived subtype composed of a relatively pure pDC population (C1) and an atypical (common)cDC-enriched subtype (C2). The latter is driven by the overexpression of DC1/2 markers *CLEC9A* and *CD1C* beyond typical pDC markers such as *CLEC4C*, *GZMB* which shape the C1 phenotype. As expected from the report by Renosi et al., we observed an additional enrichment in DC5 signatures (AS-DCs) in more than half the samples. These were, however, equally distributed across both subtypes as a putative epiphenomenon [5] (Fig. 2A). Intriguingly, genomic analysis of these newly defined subtypes revealed that C1 patients displayed a significant enrichment in *EP300*, *ARID2*, *NF1*, *NOTCH2*, and *SF3B1* mutations, whereas atypical C2 cases were enriched for *DNMT3A* and *SRSF2* mutations (Fig. 2B and Supplementary Fig. 10A). Additionally, C1 showed a significantly higher TMB (Wilcoxon test *p* = 0.002; Fig. 2C). In order to validate these observations, we performed a confirmatory multi-omics factor analysis (Fig. 2D–I; subsequent gene set enrichment see Supplementary Fig. 10B, C). A distinct clinical presentation of these subgroups, with C1 patients being significantly younger (Fisher exact test *p* = 0.0490) and C2 patients showing a trend toward lower survival, albeit in a limited subset of patients, was observed (Supplementary Fig. 11). A summary of molecular features of BPDCN derived from this study is provided in Supplementary Fig. 12. In conclusion, our multi-Omics analysis set BPDCN apart from AML whilst underscoring its close relatedness with CMML. Our findings revealed a unique molecular landscape with several novel targets and two distinct molecular signatures that advance the understanding of this entity and pave the way for precision oncology approaches toward this rare cancer entity.

**Fig. 2:**
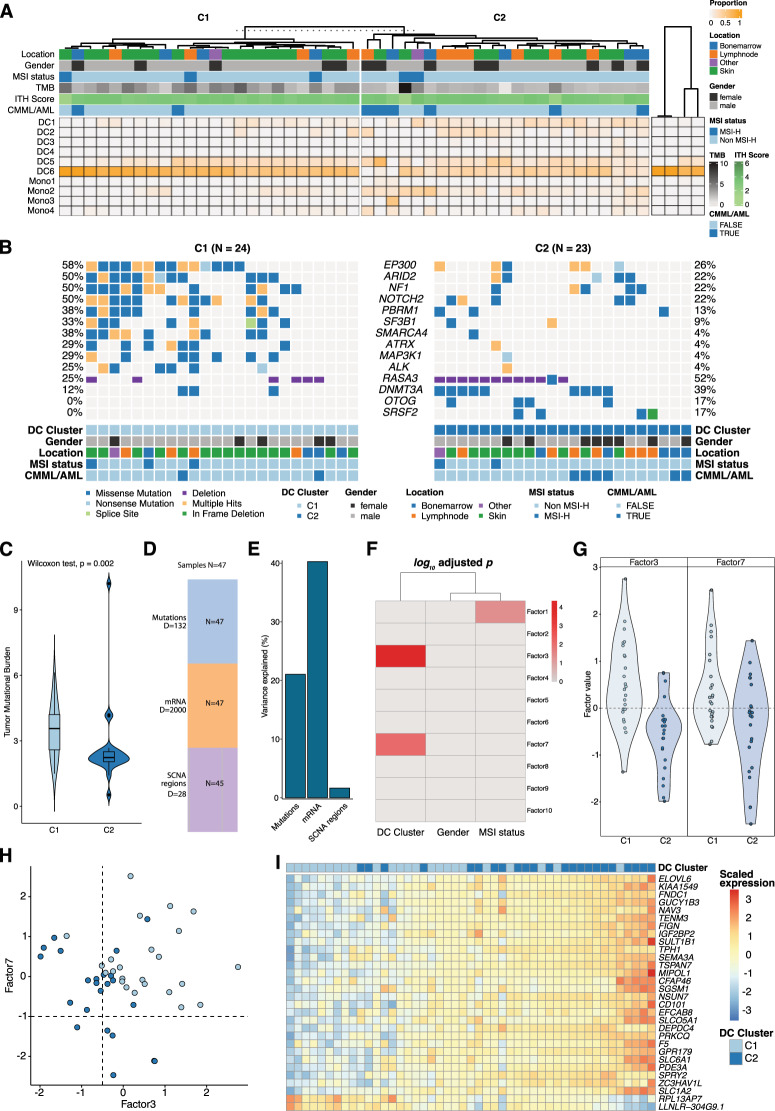
Identification of pDC and cDC-derived subtypes. **A** Proportion of dendritic cells (DC1–DC6) and monocytes (Mono1–Mono4) according to the deconvolution analysis for BPDCN samples (left heatmap with annotations; TMB refers to tumor mutational burden and ITH score describes the inferred intra-tumor heterogeneity) and normal controls (peripheral blood pDCs; shown in the right heatmap without additional annotations). The optimal number of clusters was inferred using hierarchical clustering and average silhouette method. **B** Co-oncoplot of genes identified as significantly enriched between the two cluster (see Supplementary Fig. 10 for details). **C** Tumor mutational burden estimates for each cluster. **D** shows the number of samples (“N”) and features per feature group (“D”) used in the multi-Omics factor analysis (MOFA+). **E** Variance explained per feature after training MOFA+ (Mutations = SNVs and indels; mRNA = normalized expression values; SCNA region = genomic location of somatic copy number alterations). **F** Correlation of identified factors with selected covariates (only correlations where *p*_adj_ < 0.05 are shown). **G** Beeswarm plots of latent Factor3 and Factor7 for each dendritic cell cluster. **H** Scatter plot of estimated factor values for each sample; light blue refers to C1 and dark blue to C2. **I** Scaled gene expression values of top genes (*n* = 30) that correlated with Factor3; cluster annotation for each sample is shown above each sample.

## Supplementary information

Supplemental Material

Supplemental Table 5

Supplemental Table 6

Supplemental Table 7

Supplemental Table 8

Supplemental Table 9

## Data Availability

Bam files from WXS and raw fastq files from RNA-seq have been deposited in the European genome-phenome archive (EGA) under the accession number EGAS00001006166. OncoScan Array data have been deposited in Gene Expression Omnibus (GEO) under accession number GSE200113.
