# Supplementary material for: Integrative molecular profiling identifies two molecularly and clinically distinct subtypes of blastic plasmacytoid dendritic cell neoplasm
Source: Blood Cancer J. 2022 Jul 4;12(7):101. doi: 10.1038/s41408-022-00699-1 (PMC9253119; doi:10.1038/s41408-022-00699-1)
Supplement: Supplementary file 1 — Supplemental Material [file 41408_2022_699_MOESM1_ESM.docx]

**Supplementary Materials and Methods.**

**Case selection, extraction of nucleic acids, WES and RNA-Seq**

For this retrospective analysis, we reviewed our institutional archive for cases of histologically confirmed BPDCN between January 2001 and December 2020. The study was approved by the ethics committee of the University of Lübeck (reference-no 18-311) and conducted in accordance with the declaration of Helsinki. Genomic DNA and RNA were extracted from three 5µm FFPE tissue sections of either tumor or normal tissue (where available; n = 3) employing Maxwell® RSC DNA FFPE kit and Maxwell® RSC RNA FFPE kit (both Promega). WES and RNA-Seq following library preparation using Agilent SureSelect Human All Exon V6 library preparation kit (Agilent Technologies) and NEBNext® UltraT Directional RNA Library Prep Kit (New England BioLabs), respectively were performed on a NovaSeq platform (Illumina) at Novogene (UK) Co. as described ^16^. Tumor whole exome libraries were sequenced to a median depth of 119x (mean 129 ± 54 s.d.) and normal libraries reached a median depth of 67x (mean 83 ± 36 s.d.).

**Clinicopathological assessment**

Patients at the Reference center for Hematopathology provided written informed consent regarding routine diagnostic and academic assessment, including genomic studies of their biopsy. Reassessment of histopathological diagnosis, according to the current WHO classification criteria identified 74 cases of BPDCN from which 47 cases with sufficient FFPE tissue samples for subsequent molecular analysis were selected and subjected to a comprehensive immunophenotypic workup. Antibodies and positivity cut-offs employed in the current study are summarized in **Supplementary Table 3**.

**Exome Data Processing and Variant Calling**

Raw sequencing data (fastq format) was trimmed (adapter and quality values) applying fastp^17^ (v0.23.0; minimum length, 50 bp; maximum unqualified bases, 30%; trim tail set to 1), trimmed reads were mapped to GRCh38 using bwa mem (v0.7.15)^18^ and mappings were converted into *BAM* format using Picard Tools (v2.18.4)^19^. Afterwards, mate-pair information was fixed, PCR duplicates were removed, and base quality recalibration was perform using Picard Tools, GATK (v4.2.3.0)^20^ and dbSNP v138^21^. Single nucleotide variants (SNVs) and short insertions and deletions (indels) were identified following GATKs best practices for somatic mutation calling (matched normal-tumor mode for samples with normal tissue available (n=3) and tumor-only mode for samples without normal tissue available). Briefly, Mutect2 (GATK)^22^ was applied to all *BAM* file with gnomAD variants as germline resource and the b38 exome panel from the 1000 genome project as a panel of normal, capturing the expected repertoire of germline variants to be expected in a Central European study population. Next, FFPE read orientation artifacts were identified and removed according to GATK guidelines. Filtered variants were annotated using Variant Effect Predictor (VEP v103, GRCh38; adding CADD v1.6, dbNSFP v4.1a^23^, and gnomAD r3.0 as additional annotations)^24^ and annotations were converted into *MAF* format using vcf2maf (v1.6.21) (doi:10.5281/zenodo.593251); coverage was extracted directly from the vcf INFO field. The top 20 frequently mutated genes (FLAGS)^25^ were removed from further analysis and remaining somatic variants were filtered as follows: minimum coverage of 40, minimum alternative allele coverage of 5, minimum variant allele frequency of 10%, and only variants with a frequency < 0.1% in 1000 genomes, gnomAD, or ExAC were considered for subsequent downstream analysis. High impact variants (CADD score > 10) in tumor suppressor and oncogenes according to Vogelstein *et al.* were filtered as such that a minimum coverage of 20 minimum, minimum alternative coverage and, and minimum variant allele frequency of 10% was required^26^. Genes mutated more often than expected were identified applying MutSigCV (v1.41)^27^ and potential drivers were identified suing p < 0.001. TMB did not differ between tumor-only samples and samples with matched normal tissue at finalization of. filtering. Mutational data for CMML and AML was retrieved via cBioPortal*.^28-30^*.

Selected mutations in *TET2*, *IDH1*, *RUNX1*, *ASXL1* and *TP53* in samples from which sufficient DNA was available for confirmatory investigations were subjected to either Sanger sequencing or amplicon based NGS which allowed confirmation of all investigated variants (**Supplementary Table 4**).

Coverage per sample was estimated using mosdepth (v0.3.2)^31^ and matched tumor-normal pairs were verified applying BAM-matcher^32^.

**Detection of Somatic Copy Number Alterations**

Somatic copy number alterations were detected using OncoScan CNV assays (ThermoFisher). Raw data (*CEL* files) were processed using the EaCoN package (v0.3.6-2) with SEQUENZA^33^ as segmentation algorithm. *L2R* files in CBS format were used as input for gistic (v2.0.22)^34^ to identify regions that are significantly amplified or deleted across all samples (confidence level 0.95, focal length cutoff 0.5, q-value threshold 0.1). Impact of identified regions on pathogenicity was inferred using X-CNV^35^ with standard parameters.

**Transcriptome Data Processing, Quantification, Fusion Detection, and Deconvolution**

Gene expression profiles were retrieved from adapter trimmed reads (fastp v0.23.0) using STAR aligner (v2.7.4b) against GRCh38 (gencode v37) as reference ^36^. On average 75.1 million reads (median 82.3) were successfully mapped to the human reference per tumor sample and 29.6 million reads (median 30.4) per normal sample, respectively.

Gene expression profiles were normalized applying MIXnorm (v0.0.0.9000; 50 iteration, tolerance set to 0.1)^37^, which removes unwanted biological and technical effects from formalin-fixed paraffin-embedded (FFPE) material that can bias the signal of interest. Differentially expressed genes on normalized expression values between two conditions were identified using a linear modelling approach (limma package, v3.50.1)^38^.

Pathway enrichment analysis against REACTOME gene sets (MSIGDF R package v7.2) on significantly different expressed genes was performed using a rank-MANOVA based approach as implemented in MITCH (v1.6.0; priority on significance)38; NF-𝜅β pathway (KEGG) was added manually to the REACTOME gene set.

Fusions were detected using FusionCatcher (v1.33)^39^. Identified fusions were filtered for known cancer and *MYB* fusions to remove potential false positives following the FusionCatcher guidelines. Additionally, fusion only detected by BOWTIE were discarded. Fusions were visualized using chimeraviz (v1.20.0)^40^.

Previously published scRNA-data from Villani *et al.* was used to infer the cell-type composition of the bulk RNA-Seq data with respect to dendritic cells and monocytes applying a deconvolution of damped weighted least squares (DWLS) method. Briefly, a signature matrix was built from the single cell data using default parameters (diff.cutoff = 0.5, pval.cutoff=0.01) and deconvolution was performed applying the *solveDampenedWLS* method^41,42^. The optimal number of clusters was inferred applying the average silhouette method (factoextra package v1.0.7) on deconvolution values. Xseq was employed to integrate mutational and gene expression data to assess the impact of significant gene mutations on RNA-Seq-derived profiles as described ^43^.

**Multi-omics factor analysis**

In order to underscore the molecular distinctions between the newly defined subgroups of BPDCN a confirmatory multi-omics factor analysis was performed applying an unsupervised framework (Multi-Omics Factor Analysis v2, MOFA2 v1.4.0) ^44^ ^45^ to integrate the different omics data sets (WES data, RNA-Seq data, SCNA data) to be able to discover principal sources of variation in the data. Briefly, mutated genes were considered if they were mutated in at least 4 samples selected, normalized expression values of the top 2,000 most variable genes were selected and all SCNA regions were considered for training. Feature set enrichment analysis was performed against REACTOME gene sets using parametric *t*-test on gene expression data and Benjamini-Hochberg correction to adjust p-values for multiple testing.

**Statistical analysis**

If not stated differently, statistical analysis was performed using R (v4.1.2) and p-values were corrected using Benjamini-Hochberg correction. For further details see Supplementary Material and Methods. For analysis and visualization the following R packages were applied: Tidyverse (v1.3.1)^46^ for data handling; maftools (v2.10.05)^47^ to summarize, analyze, and visualize variant data; EnhancedVolcano (v1.12.0) (https://github.com/kevinblighe/EnhancedVolcano) to show the relationship between significance and fold-changes; ComplexHeatmap (v2.10.0) and pheatmap (v1.0.12) to draw heatmaps; ggpubr (v0.4.0) for box and violin-plots; UpSetR (v1.4.0) for UpSet plots.

Progression-free survival and overall survival (PFS, OS) were calculated from the date of diagnosis and censored at last clinical contact. Survival (PFS and OS) according to potential prognostic factors was estimated by means of the Kaplan–Meier method and univariate log-rank test. Additionally, hazard ratios were calculated using a Cox proportional hazards regression model. Survival analysis was carried out employing the R packages survival (3.3-1) and survminer (v0.4.9).

Microsatellite instability was estimated with MSIseq (v1.0.0)^48^ using a custom made database of mono repeats GRCh38 (*find.mono.repeats* function) and intra-tumor heterogeneity was estimated on the entropy of somatic mutation (mDITHER-score) using DITHER (v1.0)^49^. The functional impact of mutations was assessed using xseq (v0.2.2)^43^; to estimate *trans* effects, potential *cis* effects of somatic copy number alterations were removed.

**Supplementary Table 1.** Baseline clinicopathological characteristics of the study group

| **Characteristics** | **BPDCN**  **(n = 47)** |
| --- | --- |
| **Age (yrs.; median (range))** | 74 (15 - 91) |
| **Sex** | |
| Female | 13/47 (28%) |
| Male | 34/47 (72%) |
| **Manifestation** | |
| Skin | 25/47 (53%) |
| Bone marrow | 18/47 (38%) |
| 0 EN-sites | 4/38 (11%) |
| 1-2 EN sites | 30/38 (79%) |
| > 2 EN sites | 4/38 (11%) |
| **ECOG PS** | |
| 0-1 | 16/30 (53%) |
| ≥2 | 14/30 (47%) |
| **B-symptoms** | |
| No | 12/38 (32%) |
| Yes | 26/38 (68%) |
| **LDH** | |
| Normal | 9/30 (30%) |
| Elevated | 21/30 (70%) |
| **Immunohistochemistry** | |
| BPDCN-specific | 47/47 (100%) |
| CD56^+^ | 47/47 (100%) |
| CD123^+^ | 47/47 (100%) |
| TCL1 | 40/47 (85%) |
| Immature lineage marker | 37/47 (79%) |
| CD34 | 5/47 (11%) |
| TdT^+^ | 34/47 (72%) |
| T-lineage markers | 46/47 (98%) |
| CD2^+^ | 12/41 (29%) |
| CD3^+^ | 8/47 (17%) |
| CD4^+^ | 44/47 (94%) |
| CD7^+^ | 6/47 (13%) |
| B-lineage marker CD79A^+^ | 39/47 (83%) |
| Myeloid-lineage markers | 44/47 (94%) |
| CD33^+^ | 43/47 (91%) |
| CD117^+^ | 10/47 (21%) |
| MPO^+^ | 4/47 (9%) ^#^ |
| Ki-67 (median, range) | 50% (20 – 90%) |
| **1st line therapy (available in 27 cases)** | |
| Upfront HSCT (Auto/Allo) | 6 (3/3) (22%) |
| CHOP-like regimen | 10 (37%) |
| ALL regimens* | 5 (19%) |
| Others** | 8 (30%) |
| BSC | 4 (15%) |
| Abbreviations: ALL, acute lymphoblastic leukemia; BPDCN, blastic plasmacytoid dendritic cell neoplasm; BSC, best supportive care; CHOP, cyclophosphamide/hydroxydaunorubicin/vincristine/prednisolone; ECOG, Eastern Cooperative Oncology Group; EN, extranodal; HSCT, hematopoietic stem cell transplantation; LDH, lactate dehydrogenase; MPO, myeloperoxidase; yrs, years.  *GMALL, B-ALL protocol  **DeVIC protocol (n=2), azazitidine-mono (n=4), radiation therapy (n=1), DHAC (n=1)  ^#^ second, smaller population, positive for CD123 and MPO | |

**Supplementary Table 2.** Immunohistochemical profile of the study cohort

| **Case IDN-Nr.** | **localization** | **CD3** | **CD4** | **CD33** | **CD34** | **CD56** | **CD79a** | **CD117** | **CD123** | **ASD** | **Lyso** | **Ki67** | **MPO** | **PGM1** | **TCL1** | **TdT** |
| --- | --- | --- | --- | --- | --- | --- | --- | --- | --- | --- | --- | --- | --- | --- | --- | --- |
| BPDCN_01 | lymph node | - | + | +/- | - | + | - | - | + | - | - | 60% | - | - | + | +/- |
| BPDCN_02 | skin | +/- | + | + | - | + | +/- | - | + | - | - | 30% | - | +/- | + | +/- |
| BPDCN_05 | bone marrow | - | + | - | - | + | - | - | + | - | - | 90% | - | - | + | - |
| BPDCN_06 | lymph node | - | - | + | - | + | +/- | +/- | + | - | - | 60% | - | - | + | + |
| BPDCN_07 | skin | - | + | +/- | - | + | - | - | + | - | - | 60% | - | - | + | + |
| BPDCN_08 | skin | - | +/- | +/- | - | + | +/- | - | + | - | - | 30% | - | +/- | + | + |
| BPDCN_09 | skin | - | + | +/- | - | + | + | - | + | - | - | 80% | - | - | +/- | +/- |
| BPDCN_10 | lymph node | - | - | + | - | + | +/- | +/- | + | - | - | 25% | - | - | + | +/- |
| BPDCN_11 | skin | - | + | + | +/- | +/- | +/- | - | + | - | - | 30% | - | + | + | + |
| BPDCN_12 | bone marrow | - | + | +/- | - | + | +/- | - | + | - | - | 30% | - | +/- | + | +/- |
| BPDCN_13 | skin | - | + | + | - | + | - | - | + | - | - | 20% | - | - | + | - |
| BPDCN_14 | skin | - | + | + | - | + | - | - | + | - | - | 50% | - | - | + | +/- |
| BPDCN_15 | lymph node | - | +/- | +/- | +/- | +/- | - | +/- | + | - | - | 40% | +/- | - | - | +/- |
| BPDCN_16 | skin | - | + | + | - | + | - | - | + | - | - | 50% | - | + | - | - |
| BPDCN_17 | skin | - | + | +/- | - | + | +/- | - | + | - | - | 50% | - | - | + | + |
| BPDCN_18 | skin | - | +/- | + | - | + | +/- | - | + | - | - | 70% | - | +/- | + | - |
| BPDCN_19 | fatty tissue | +/- | + | - | - | + | + | - | + | - | - | 90% | - | - | - | - |
| BPDCN_20 | skin | - | + | + | - | + | +/- | - | + | - | - | 30% | - | - | + | +/- |
| BPDCN_21 | skin | - | + | +/- | - | + | +/- | - | + | - | - | 30% | - | - | + | +/- |
| BPDCN_22 | lymph node | - | +/- | +/- | - | + | +/- | - | + | - | - | 30% | - | - | + | - |
| BPDCN_23 | skin | - | + | +/- | - | + | + | - | + | - | - | 30% | - | +/- | + | + |
| BPDCN_24 | skin | +/- | + | - | - | + | +/- | - | + | - | - | 70% | - | - | + | +/- |
| BPDCN_25 | lymph node | +/- | - | + | + | +/- | + | +/- | + | - | +/- | 60% | +/- | +/- | - | +/- |
| BPDCN_26 | skin | - | + | + | - | + | + | - | + | - | - | 30% | - | - | + | +/- |
| BPDCN_27 | skin | - | +/- | + | - | + | + | - | + | +/- | + | 60% | - | + | - | - |
| BPDCN_28 | mucosa | - | +/- | +/- | - | + | +/- | - | + | - | - | 60% | - | - | + | + |
| BPDCN_29 | lymph node | - | + | +/- | - | + | +/- | +/- | + | - | - | 30% | +/- | - | + | + |
| BPDCN_30 | lymph node | - | + | + | - | + | + | +/- | + | - | +/- | 60% | - | + | + | +/- |
| BPDCN_31 | skin/mucosa | - | + | + | - | + | +/- | +/- | + | - | - | 60% | - | - | +/- | - |
| BPDCN_32 | lymph node | - | +/- | +/- | - | + | +/- | +/- | + | - | +/- | 30% | - | - | + | +/- |
| BPDCN_33 | skin | - | + | +/- | - | + | +/- | - | + | - | - | 30% | - | - | + | - |
| BPDCN_34 | bone marrow | - | + | +/- | - | + | +/- | +/- | + | - | - | 40% | - | + | + | +/- |
| BPDCN_36 | skin | - | +/- | + | - | + | +/- | +/- | + | - | +/- | 30% | - | - | + | + |
| BPDCN_37 | bone marrow | - | + | - | + | + | - | - | + | +/- | + | 60% | +/- | + | - | - |
| BPDCN_38 | bone marrow | +/- | + | +/- | - | + | + | - | + | - | - | 60% | - | - | + | - |
| BPDCN_40 | bone marrow | - | + | +/- | - | + | + | - | + | - | - | 80% | - | - | +/- | - |
| BPDCN_41 | skin | - | + | + | - | + | +/- | - | + | - | - | 70% | - | +/- | + | - |
| BPDCN_42 | lymph node | - | + | + | - | + | +/- | - | + | - | - | 60% | - | +/- | + | - |
| BPDCN_43 | bone marrow | +/- | + | + | - | + | +/- | - | + | - | - | 50% | - | - | + | +/- |
| BPDCN_44 | bone marrow | - | + | + | - | + | +/- | - | + | - | - | 60% | - | - | + | + |
| BPDCN_45 | bone marrow | +/- | +/- | + | + | + | +/- | +/- | + | - | - | 50% | - | + | - | - |
| BPDCN_46 | skin | - | + | + | - | + | + | - | + | - | - | 30% | - | - | + | + |
| BPDCN_47 | skin | - | + | + | - | + | +/- | - | + | - | - | 40% | - | - | + | +/- |
| BPDCN_48 | lymph node | - | + | + | - | + | + | - | + | - | - | 30% | - | + | + | +/- |
| BPDCN_49 | skin | - | + | + | - | + | + | - | + | - | - | 70% | - | + | + | +/- |
| BPDCN_50 | skin | - | + | + | - | + | +/- | - | + | - | - | 80% | - | - | + | +/- |
| BPDCN_51 | skin | +/- | + | + | - | + | +/- | - | + | - | - | 50% | - | + | + | +/- |
| +; > 60 % of nucleated cells/surface area. +/-; 10 - 60 % of nucleated cells/surface area. -; < 10% of nucleated cells/surface area | | | | | | | | | | | | | | | | |

|  |  |
| --- | --- |

**Supplementary Table 3.** Antibodies and conditions used throughout the study

| **Antibody** | **Manufacturer** | **Clone** | **Dilution** | **Incubation period** |
| --- | --- | --- | --- | --- |
| CD3 | Leica | LN10 | RTU | 30min |
| CD4 | Leica | 4B12 | RTU | 30min |
| CD33 | Leica | PWS44 | RTU | 30min |
| CD34 | Leica | Qdend | RTU | 30min |
| CD56 | Leica | CD564 | RTU | 30min |
| CD79a | Leica | JCB117 | RTU | 30min |
| CD117 | leica | EP10 | RTU | 30min |
| CD123 | Leica | BR4MS | 1:100 | 30min |
| Lysozym | DAKO Agilent | A 0099 | 1:10000 | 30min |
| Ki67 | Leica | K2 | RTU | 30min |
| MPO | Leica | 59A5 | RTU | 30min |
| CD068 | DAKO Agilent | PG-M1 | 1:100 | 30min |
| TCL-1 | Menarini | MRQ-7 | 1:100 | 30min |
| Tdt | Leica | SEN28 | RTU | 30min |
| RTU, ready to use dilution | | | | |

**Supplementary Table 4.** Confirmatory Sanger and amplicon-based next generation sequencing of selected variants

| **CaseID** | **Chromosome** | **Reference_Allele** | **Alt_Allele** | **Hugo_Symbol** | **CDS_position** | **AAChange** | **vaf** | **AAPos** | **Methode** |
| --- | --- | --- | --- | --- | --- | --- | --- | --- | --- |
| BPDCN_05 | chr17 | C | T | TP53 | 524 | TP53:p.R175H | 0,4746 | 175 | NGS |
| BPDCN_10 | chr17 | T | C | TP53 | 488 | TP53:p.Y163C | 0,3872 | 163 | NGS |
| BPDCN_15 | chr21 | C | A | RUNX1 | 611 | RUNX1:p.R204L | 0,5082 | 204 | NGS |
| BPDCN_21 | chr4 | G | A | TET2 | 3886 | TET2:p.G1296R | 0,4364 | 1296 | NGS |
| BPDCN_25 | chr2 | G | A | IDH1 | 394 | IDH1:p.R132C | 0,4636 | 132 | NGS |
| BPDCN_26 | chr4 | C | T | TET2 | 1858 | TET2:p.Q620* | 0,3874 | 620 | Sanger |
| BPDCN_45 | chr4 | A | T | TET2 | 2314 | TET2:p.K772* | 0,2741 | 772 | Sanger |
| BPDCN_49 | chr20 | C | T | ASXL1 | 1762 | ASXL1:p.Q588* | 0,3182 | 588 | Sanger |
| BPDCN_49 | chr4 | C | T | TET2 | 2431 | TET2:p.Q811* | 0,2698 | 811 | Sanger |

**Supplementary Table 5.** MutSigCV analysis. Separate Excel file.

**Supplementary Table 6.** Identified variants per sample and predicted functional impact. Separate Excel file.

**Supplementary Table 7.** SCNA results by gistic analysis (losses) and functional impact assessment. Separate Excel file.

**Supplementary Table 8.** SCNA results by GISTIC analysis (gains) and functional impact assessment. Separate Excel file.

**Supplementary Table 9.** Fusions. Separate Excel file.

**Supplementary Figures**

**Supplementary Figure 1.** Pathway-enrichment analysis.

**Supplementary Figure 2** (A) Spatial clustering of mutations within the protein structure of NRAS (PDB ID 6E6H) and (B) eGFR (PDB ID 6TG0).

**Supplementary Figure 3.** (A) Mutual exclusive occurrence of *NRAS* with *CIC* and *MET*; type of mutation is color coded. (B)Combinations of mutational (exclusive) co-occurrences.

**Supplementary Figure 4.** Age dependent mutated genes.

**Supplementary Figure 5.** (A) Tumor suppressor genes affected by copy number losses (light blue: low level deletions; dark blue: high level deletions) and (B) oncogenes affected by copy number gains (light red: low level amplifications; dark red: high level amplifications).


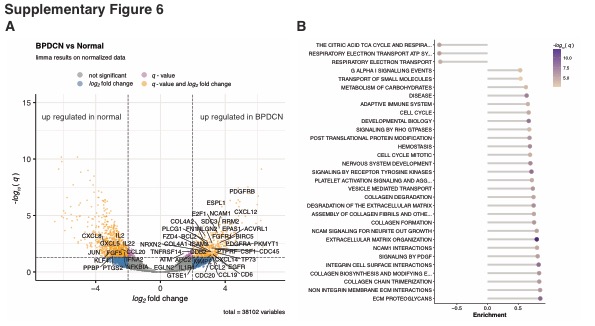


**Supplementary Figure 6. Patterns of differentially expressed genes in BPDCN** (A) Volcano plot showing *log_2_* fold-changes and *-log_10_* scaled q-values of the expression analysis of BPDCN samples against normal controls (peripheral blood pDCs). Dotted lines denote significance level (q < 0.05) and absolute *log_2_* fold-changes above 2; known tumor suppressor genes, oncogenes and genes related to cytokine-cytokine receptor interaction, cell adhesion molecules cams, cell cycle, P53 signaling pathway and pathways in cancer (all retrieved from KEGG) are highlighted if they are significant. (B) Pathway enrichment analysis against REACTOME gene sets on differentially expressed genes (q < 0.01, absolute *log_2_* fold-change > 2); enrichment scores (length of bar) and *-log_10_* scaled q-values (color-coded dots) are displayed for pathways with q < 0.05 and absolute enrichment scores > 0.5.

**Supplementary Figure 7.** Panels (A-F) show significant differences in expression levels of selected genes in regions of copy number losses.

**Supplementary** **Figure 8. *trans* effects of mutations on gene expression.** Significant *trans* effects of mutations on gene expression for *TP53*, *RUNX1*, and *CBL* identified applying xseq; red represents high expression and blue represents low expression; P(F) denote probability values are color coded; type of mutation is color coded.

**
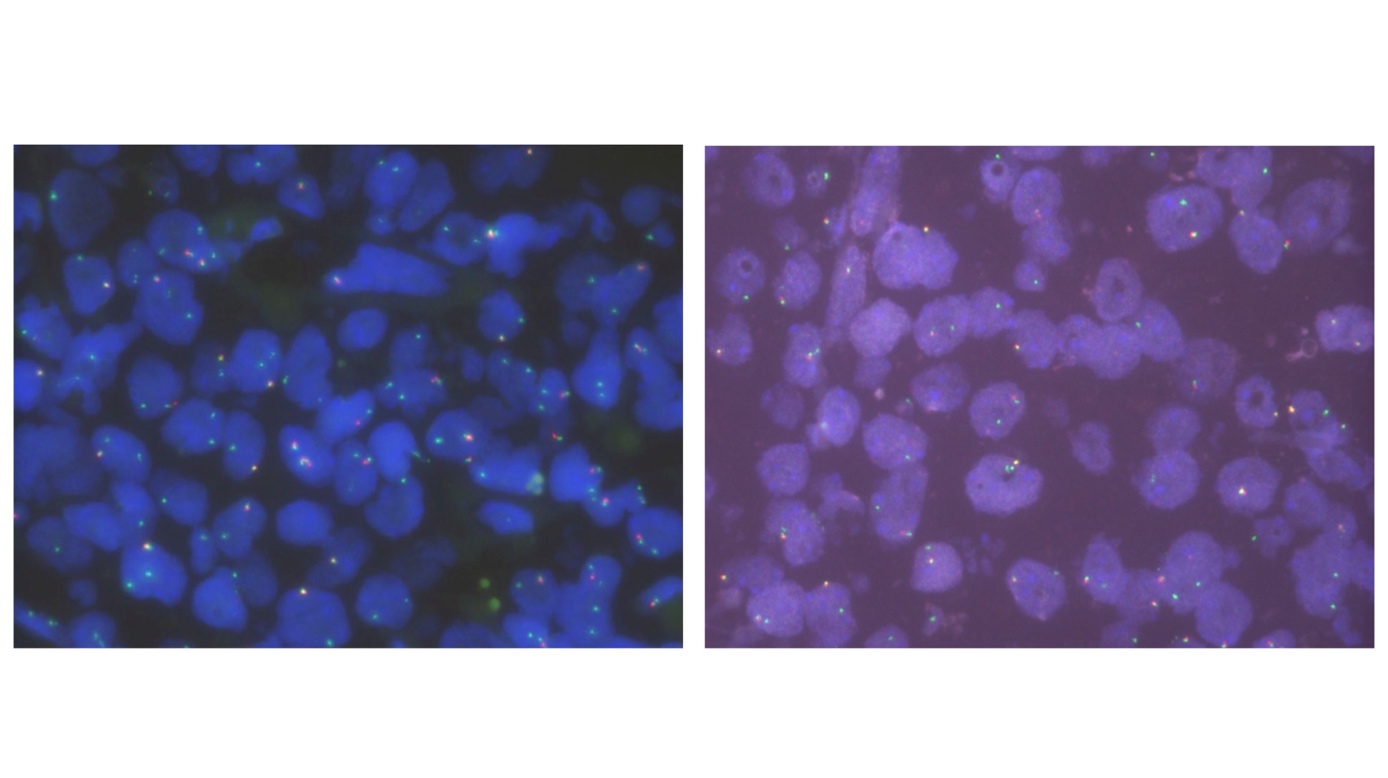
**

**Supplementary Figure 9.** FISH validation of two representative cases harboring a *MYB* fusion according to RNA-seq.

**Supplementary Figure 10.** (A) DC-Cluster dependent distribution of mutations. (B) Feature set enrichment of positive and (C) of negative weights for Factor3.

**
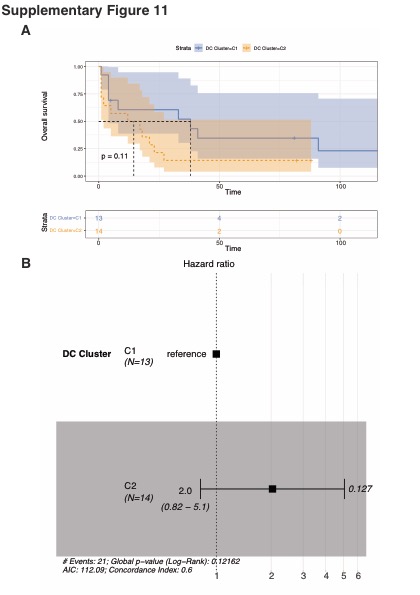
**

**Supplementary Figure 11.** (A) Overall survival by Kaplan-Meier and (B) Hazard-ratio according to DC-Cluster.

**
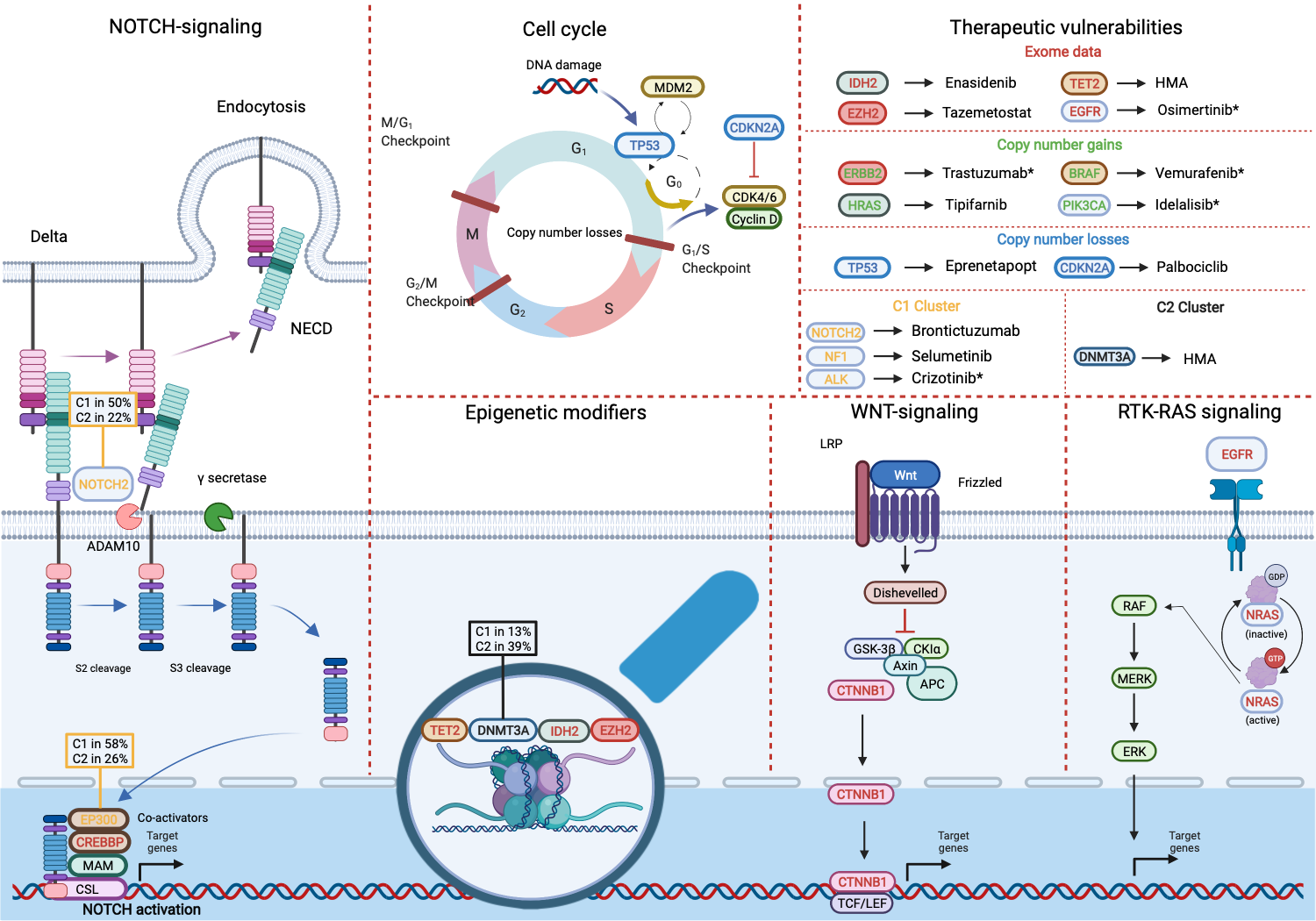
**

**Supplementary** **Figure 12. Visualization of predominantly altered pathways and potential therapeutic targets in BPDCN.** Whole exome sequencing (WES) revealed frequent alterations NOTCH (*NOTCH2, CREBBP, EP300*), WNT (*CTNNB1*) and RTK-RAS (*EGFR, NRAS*) signaling as well as in epigenetic modifiers (*TET2, DNMT3A, EZH2* and *IDH2*). Additionally, SCNA analysis identified two copy number losses (*TP53* and *CDKN2A*) significantly affecting cell cycle regulation towards cancer progression. Integrated WES/RNA-seq analysis divided two distinct BPDCN subtypes in accordance with a subtype-specific mutational signature. The C1 cluster harbours *NOTCH2*, *EP300*, *NF1* and *ALK* mutations whereas *DNMT3A* mutations were found to be recurrent features within the C2 cluster. Further, therapeutically targetable vulnerabilities uncovered by comprehensive genomic profiling are summarized.

**Supplementary References**

16. Witte HM, Kunstner A, Hertel N, et al. Integrative genomic and transcriptomic analysis in plasmablastic lymphoma identifies disruption of key regulatory pathways. *Blood Adv*. 2021.

17. Chen S, Zhou Y, Chen Y, Gu J. fastp: an ultra-fast all-in-one FASTQ preprocessor. *Bioinformatics*. 2018;34(17):i884-i890.

18. Li H. Aligning sequence reads, clone sequences and assembly contigs with BWA-MEM; 2013:arXiv:1303.3997.

19. Institute B. Picard Toolkit. GitHub Repository; 2019.

20. McKenna A, Hanna M, Banks E, et al. The Genome Analysis Toolkit: a MapReduce framework for analyzing next-generation DNA sequencing data. *Genome Res*. 2010;20(9):1297-1303.

21. Sherry ST, Ward MH, Kholodov M, et al. dbSNP: the NCBI database of genetic variation. *Nucleic Acids Res*. 2001;29(1):308-311.

22. Cibulskis K, Lawrence MS, Carter SL, et al. Sensitive detection of somatic point mutations in impure and heterogeneous cancer samples. *Nat Biotechnol*. 2013;31(3):213-219.

23. Rentzsch P, Witten D, Cooper GM, Shendure J, Kircher M. CADD: predicting the deleteriousness of variants throughout the human genome. *Nucleic Acids Res*. 2019;47(D1):D886-D894.

24. McLaren W, Gil L, Hunt SE, et al. The Ensembl Variant Effect Predictor. *Genome Biol*. 2016;17(1):122.

25. Shyr C, Tarailo-Graovac M, Gottlieb M, Lee JJ, van Karnebeek C, Wasserman WW. FLAGS, frequently mutated genes in public exomes. *BMC Med Genomics*. 2014;7:64.

26. Vogelstein B, Papadopoulos N, Velculescu VE, Zhou S, Diaz LA, Jr., Kinzler KW. Cancer genome landscapes. *Science*. 2013;339(6127):1546-1558.

27. Lawrence MS, Stojanov P, Polak P, et al. Mutational heterogeneity in cancer and the search for new cancer-associated genes. *Nature*. 2013;499(7457):214-218.

28. Papaemmanuil E, Gerstung M, Malcovati L, et al. Clinical and biological implications of driver mutations in myelodysplastic syndromes. *Blood*. 2013;122(22):3616-3627; quiz 3699.

29. Papaemmanuil E, Gerstung M, Bullinger L, et al. Genomic Classification and Prognosis in Acute Myeloid Leukemia. *N Engl J Med*. 2016;374(23):2209-2221.

30. Tyner JW, Tognon CE, Bottomly D, et al. Functional genomic landscape of acute myeloid leukaemia. *Nature*. 2018;562(7728):526-531.

31. Pedersen BS, Quinlan AR. Mosdepth: quick coverage calculation for genomes and exomes. *Bioinformatics*. 2018;34(5):867-868.

32. Wang PP, Parker WT, Branford S, Schreiber AW. BAM-matcher: a tool for rapid NGS sample matching. *Bioinformatics*. 2016;32(17):2699-2701.

33. Favero F, Joshi T, Marquard AM, et al. Sequenza: allele-specific copy number and mutation profiles from tumor sequencing data. *Ann Oncol*. 2015;26(1):64-70.

34. Mermel CH, Schumacher SE, Hill B, Meyerson ML, Beroukhim R, Getz G. GISTIC2.0 facilitates sensitive and confident localization of the targets of focal somatic copy-number alteration in human cancers. *Genome Biol*. 2011;12(4):R41.

35. Zhang L, Shi J, Ouyang J, et al. X-CNV: genome-wide prediction of the pathogenicity of copy number variations. *Genome Med*. 2021;13(1):132.

36. Dobin A, Davis CA, Schlesinger F, et al. STAR: ultrafast universal RNA-seq aligner. *Bioinformatics*. 2013;29(1):15-21.

37. Yin S, Wang X, Jia G, Xie Y. MIXnorm: normalizing RNA-seq data from formalin-fixed paraffin-embedded samples. *Bioinformatics*. 2020;36(11):3401-3408.

38. Ritchie ME, Phipson B, Wu D, et al. limma powers differential expression analyses for RNA-sequencing and microarray studies. *Nucleic Acids Res*. 2015;43(7):e47.

39. Nicorici D, Şatalan M, Edgren H, et al. <strong>FusionCatcher</strong> – a tool for finding somatic fusion genes in paired-end RNA-sequencing data. *bioRxiv*. 2014:011650.

40. Lagstad S, Zhao S, Hoff AM, Johannessen B, Lingjaerde OC, Skotheim RI. chimeraviz: a tool for visualizing chimeric RNA. *Bioinformatics*. 2017;33(18):2954-2956.

41. Villani AC, Satija R, Reynolds G, et al. Single-cell RNA-seq reveals new types of human blood dendritic cells, monocytes, and progenitors. *Science*. 2017;356(6335).

42. Tsoucas D, Dong R, Chen H, Zhu Q, Guo G, Yuan GC. Accurate estimation of cell-type composition from gene expression data. *Nat Commun*. 2019;10(1):2975.

43. Ding J, McConechy MK, Horlings HM, et al. Systematic analysis of somatic mutations impacting gene expression in 12 tumour types. *Nat Commun*. 2015;6:8554.

44. Argelaguet R, Velten B, Arnol D, et al. Multi-Omics Factor Analysis-a framework for unsupervised integration of multi-omics data sets. *Mol Syst Biol*. 2018;14(6):e8124.

45. Argelaguet R, Arnol D, Bredikhin D, et al. MOFA+: a statistical framework for comprehensive integration of multi-modal single-cell data. *Genome Biol*. 2020;21(1):111.

46. Wickham H, Averick M, Bryan J, et al. Welcome to the Tidyverse. *Journal of Open Source Software*. 2019;4(43).

47. Mayakonda A, Lin DC, Assenov Y, Plass C, Koeffler HP. Maftools: efficient and comprehensive analysis of somatic variants in cancer. *Genome Res*. 2018;28(11):1747-1756.

48. Huang MN, McPherson JR, Cutcutache I, Teh BT, Tan P, Rozen SG. MSIseq: Software for Assessing Microsatellite Instability from Catalogs of Somatic Mutations. *Sci Rep*. 2015;5:13321.

49. Li L, Chen C, Wang X. DITHER: an algorithm for Defining IntraTumor Heterogeneity based on EntRopy. *Brief Bioinform*. 2021;22(6).
